# Safety and immunogenicity of a modified COVID-19 mRNA vaccine, SYS6006, as a fourth-dose booster following three doses of inactivated vaccines in healthy adults: an open-labeled Phase 1 trial

**DOI:** 10.1093/lifemeta/load019

**Published:** 2023-05-10

**Authors:** Yuzhou Gui, Ye Cao, Jiajin He, Chunyang Zhao, Wei Zheng, Ling Qian, Jie Cheng, Chengyin Yu, Chen Yu, Kun Lou, Gangyi Liu, Jingying Jia

**Affiliations:** Shanghai Xuhui Central Hospital/Xuhui Hospital, Fudan University, Shanghai 200031, China; Shanghai Engineering Research Center of Phase I Clinical Research & Quality Consistency Evaluation for Drugs, Shanghai 200031, China; Shanghai Xuhui Central Hospital/Xuhui Hospital, Fudan University, Shanghai 200031, China; Shanghai Engineering Research Center of Phase I Clinical Research & Quality Consistency Evaluation for Drugs, Shanghai 200031, China; Shanghai Xuhui Central Hospital/Xuhui Hospital, Fudan University, Shanghai 200031, China; Shanghai Engineering Research Center of Phase I Clinical Research & Quality Consistency Evaluation for Drugs, Shanghai 200031, China; Shanghai Xuhui Central Hospital/Xuhui Hospital, Fudan University, Shanghai 200031, China; Shanghai Engineering Research Center of Phase I Clinical Research & Quality Consistency Evaluation for Drugs, Shanghai 200031, China; Shanghai Xuhui Central Hospital/Xuhui Hospital, Fudan University, Shanghai 200031, China; Shanghai Engineering Research Center of Phase I Clinical Research & Quality Consistency Evaluation for Drugs, Shanghai 200031, China; Shanghai Xuhui Central Hospital/Xuhui Hospital, Fudan University, Shanghai 200031, China; Shanghai Engineering Research Center of Phase I Clinical Research & Quality Consistency Evaluation for Drugs, Shanghai 200031, China; Shanghai Xuhui Central Hospital/Xuhui Hospital, Fudan University, Shanghai 200031, China; Shanghai Engineering Research Center of Phase I Clinical Research & Quality Consistency Evaluation for Drugs, Shanghai 200031, China; Shanghai Xuhui Central Hospital/Xuhui Hospital, Fudan University, Shanghai 200031, China; Shanghai Engineering Research Center of Phase I Clinical Research & Quality Consistency Evaluation for Drugs, Shanghai 200031, China; Shanghai Xuhui Central Hospital/Xuhui Hospital, Fudan University, Shanghai 200031, China; Shanghai Engineering Research Center of Phase I Clinical Research & Quality Consistency Evaluation for Drugs, Shanghai 200031, China; CSPC Zhongqi Pharmaceutical Technology (Shijiazhuang) Co., Ltd, Shijiazhuang, Hebei 050035, China; Shanghai Xuhui Central Hospital/Xuhui Hospital, Fudan University, Shanghai 200031, China; Shanghai Engineering Research Center of Phase I Clinical Research & Quality Consistency Evaluation for Drugs, Shanghai 200031, China; Shanghai Xuhui Central Hospital/Xuhui Hospital, Fudan University, Shanghai 200031, China; Shanghai Engineering Research Center of Phase I Clinical Research & Quality Consistency Evaluation for Drugs, Shanghai 200031, China

**Keywords:** SARS-CoV-2, mRNA vaccine, SYS6006, heterologous boosting, clinical trial

## Abstract

The continuous emergence of the severe acute respiratory syndrome coronavirus 2 (SARS-CoV-2) variants led to a rapid decline in protection efficacy and neutralizing titers even after three doses of COVID-19 vaccines. Here, we report an open-labeled Phase I clinical trial of a modified mRNA vaccine (SYS6006) as a fourth-dose booster in healthy adults. Eighteen eligible participants, who had completed three doses of inactivated COVID-19 vaccines, received a fourth boosting dose of SYS6006-20 μg. Eighteen convalescent COVID-19 patients were enrolled for the collection of serum samples as a comparator of immunogenicity. The primary endpoint of this trial was titers of anti-receptor binding domain of spike glycoprotein (RBD) antibodies of the Omicron strain (BA.2 and BA.4/5) in serum; titers of neutralizing antibodies against pseudovirus of the Omicron strain (BA.2 and BA.4/5). The secondary endpoint was the incidence of adverse events within 30 days after the boosting. The exploratory endpoint was the cellular immune responses (interferon gamma, IFN-γ). This trial was registered with the Chinese Clinical Trial Registry website. No serious adverse events were reported within 30 days after vaccination. No Grade 3 fever or serious adverse event was reported in the SYS6006 group. Notably, SYS6006 elicited higher titers and longer increases in anti-RBD antibodies and neutralizing antibodies (>90 days) compared with the convalescent group (*P* < 0.0001) against Omicron strain (BA.2 and BA.4/5). Besides, higher positive spots of T-cell-secreting IFN-γ were observed in the SYS6006 group than those in the convalescent group (*P* < 0.05). These data demonstrated that SYS6006 was well tolerated and highly immunogenic, generating a stronger and more durable immune response against different variants of SARS-CoV-2.

## Introduction

The COVID-19 pandemic, caused by severe acute respiratory syndrome coronavirus 2 (SARS-CoV-2), has resulted in more than 600 million confirmed cases and 6.5 million deaths worldwide [1]. A remarkable global effort has been made to develop vaccines by using a variety of traditional and innovative platforms, such as inactivated viruses, messenger ribonucleic acid (mRNA), deoxyribonucleic acid, live viral vectors, recombinant proteins, and peptides [2]. Currently, 175 of these vaccine candidates have entered clinical development and at least 50 of them have been approved for use in at least one country [3]. Despite the vaccine campaign around the world for control and prevention of COVID-19 cases, the declining efficacy against the emergence of the variants of SARS-CoV-2 calls for additional safe and effective vaccines [4].

The boosting dose of the COVID-19 vaccine was required due to waning immunity and the possibility of continued circulation of additional SARS-CoV-2 variants, even though protection against infection and mild disease appeared to wane in the months following vaccination [5–8]. Recent studies have shown that a heterologous schedule produces a much stronger and longer immune response than homologous boosting vaccination [9–13]. Moreover, the World Health Organization suggests that heterologous vaccination with vector vaccines or mRNA vaccines has better immunogenicity and a higher level of neutralizing antibodies than homologous booster vaccination with inactivated vaccines, based on the evidence that is currently available [14]. More importantly, a heterologous booster is more effective in preventing COVID-19 infection than a primary two-dose series of inactivated vaccines [15]. In a small group of pivotal trial participants, a third BNT162b2 dose given 7–9 months after the primary two-dose series increased the magnitude and breadth of the immune response [16]. Moreover, US Food and Drug Administration authorized the second booster shot of the Pfizer BioNTech or Moderna vaccine, which is the fourth dose of the COVID-19 vaccine for high-risk groups of people, including the elderly. Researchers have observed significantly higher neutralizing antibodies and efficacy after the fourth dose of mRNA-1273 during the Omicron epidemic [17].

Humoral immunity is substantially linked with protection against SARS-CoV-2 infection [18]. Both binding and neutralizing antibody-mediated viral neutralization is frequently regarded as a measure of protection [19, 20]. Parallel to this, cell-mediated immunity, including T-cell-mediated cytokine secretion, has been proposed to be more cross-protective among emerging viruses of concern (VOCs) and contribute to viral clearance, serious illness prevention, and long-term immunity [21–23]. Since convalescent patients have immunity against the same virus strain, the serum specimen of convalescent patients is used as a comparator for evaluating the efficacy and duration of an investigational vaccine [24, 25].

mRNA-based vaccines have emerged as a leading platform for COVID-19 protection and are extensively investigated in basic and clinical trials [26–28]. It can induce comparable high spike-specific binding concentrations for a variety of newly developing VOCs. Yet, the boosting regimen of COVID-19 mRNA vaccines is not available in China. Moreover, the third dose of inactivated vaccine of COVID-19 has been proposed as the initial boosting regimen, and the second booster (the fourth dose of the vaccine) is worth investigating [29–31]. SYS6006 (CSPC Pharmaceutical Group) is a newly investigational COVID-19 mRNA vaccine encoding a full-length S protein sequence of the prototype SARS-CoV-2 strain and incorporating the key mutations of main epidemic variants. To enhance immunogenicity, K986P and V987P mutations (S-2P) are also introduced to maintain the prefusion conformation of the encoded antigen. The results of preclinical studies showed that after inoculation with SYS6006 in mice and non-human primates, higher titers of neutralizing antibodies and conjugated antibodies could be produced, which could protect mice from pathological injury and death caused by the attack of the new coronavirus Delta and Omicron variants, and had a good safety profile [32]. It has been approved for clinical development and currently, four clinical trials have been completed. In March 2023, it has been authorized for emergent use in China by the national medicinal product agency of China.

Since the inactivated vaccines are applied widely in the vaccine campaign for COVID-19, it is worth investigating the heterologous boosting effect of the mRNA vaccine. Here, we provide the evaluation of the safety and immunogenicity results of a modified COVID-19 mRNA, SYS6006, as a fourth-dose booster in healthy Chinese adults. The humoral and cellular immune response of boosting vaccination is assessed with the results of convalescent samples as the comparator.

## Results

### Demographic and baseline clinical characteristics

**Figure 1:**
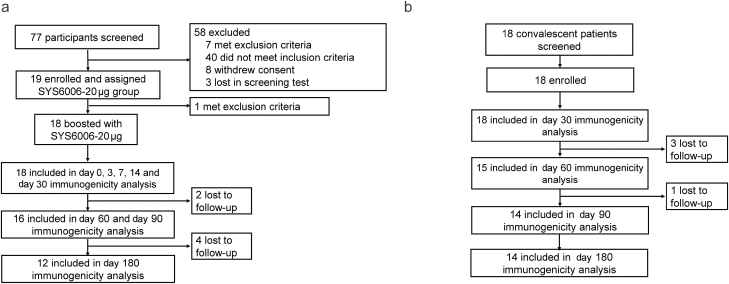
Trial profile. (a) SYS6006 group; (b) Convalescent group.

Between 7 July and 19 July 2022, 77 adults aged between 18 and 70 years were screened at Shanghai Xuhui Central Hospital. Of them, 19 participants, who had received three doses of inactivated COVID-19 vaccine for more than 6 months, were enrolled and received one dose of SYS6006-20 μg (Group A). One participant met the exclusion criteria before receiving the vaccination. Finally, 18 participants received one dose of SYS6006-20 μg (Group A). For Group B, 18 convalescent COVID-19 patients were enrolled for the collection of serum samples (Fig. 1). Convalescent patients were in the cabin hospitals and were not able to complete the blood drawing process at day 14 and 21 post COVID-19 infection. The demographic characteristics of participants were listed in Table 1. The baseline demographic characteristics of the participants at enrollment were similar between Group A and Group B in terms of sex, median age, and ethnic group. The time interval between the third dose and the fourth dose of vaccination was 218 days for Group A participants. The vaccination history and the strain of SARS-CoV-2 were unable to retrieve. The strain, that the convalescent group was infected with, was presumably Omicron BA.2 according to the reports and reference [33].

**Table 1:** Baseline demographic characteristics.

	SYS6006-20 μg booster group (*n* = 18)	Convalescent patients (*n* = 18)
**Sex**		
Male	15 (83.3%)	15 (83.3%)
Female	3 (16.7%)	3 (16.7%)
**Age, years**	31.6 (19−44)	31 (19−45)
**BMI, kg/m** ^**2**^	22.7 (19.5−27.4)	-
**Ethnicity**		
Han	17 (94.4%)	17 (94.4%)
Others	1 (5.6%)	1 (5.6%)
**Vaccination history (three doses)**		
COVILO	10 (55.6%)	/
CoronaVac	8 (44.4%)	/
**Time interval between the primary series and the booster dose**		
Median (IQR, day)	218 (189−279)	-

Data are *n* (%), mean (SD), or median (IQR).

### Safety and tolerability

Fifteen out of 18 (83.3%) participants in Group A reported solicited adverse events. Among them, injection site pain was the most common adverse event (15/18, 83.3%). The systemic adverse events after the booster doses were fever, headache, fatigue, nausea, or cough (Table 2) in the SYS6006-20 μg group (Group A). The most common systemic adverse event was fever, which occurred in 7 of 18 participants (38.9%) in Group A. No Grade 3 adverse events were reported. All solicited adverse events were mild (Grade 1) or moderate (Grade 2), which were managed with a simple standard of care or resolved spontaneously. Nine out of 18 (50.0%) participants in the SYS6006 group reported unsolicited adverse events within 30 days after vaccination. The most common unsolicited adverse event was the increase of urine red cells (4/18, 22.2%). No Grade 3 unsolicited adverse events were reported.

**Table 2:** Solicited adverse events that occurred in the SYS6006 booster group (*n* = 18) within 14 days after vaccination, graded by NMPA criteria.

Solicited adverse events	
Any	15 (83.3%)
Grade 3	0 (0%)
Injection site adverse reactions	
Any	15 (83.3%)
Grade 3	0 (0%)
Pain	15 (83.3%)
Pruritus	1 (5.6%)
Redness	1 (5.6%)
Swelling	2 (11.1%)
Systemic adverse reactions	
Any	8 (44.4%)
Grade 3	0 (0%)
Fever	7 (38.9%)
Headache	1 5.6%)
Fatigue or malaise	1 (5.6%)
Nausea	1 (5.6%)
Cough	1 (5.6%)

Data are *n* (%), *n*, the number of participants; %, the proportion of participants.

### Immunogenicity assessments

#### Receptor binding domain of spike glycoprotein (RBD)-specific antibodies

**Figure 2:**
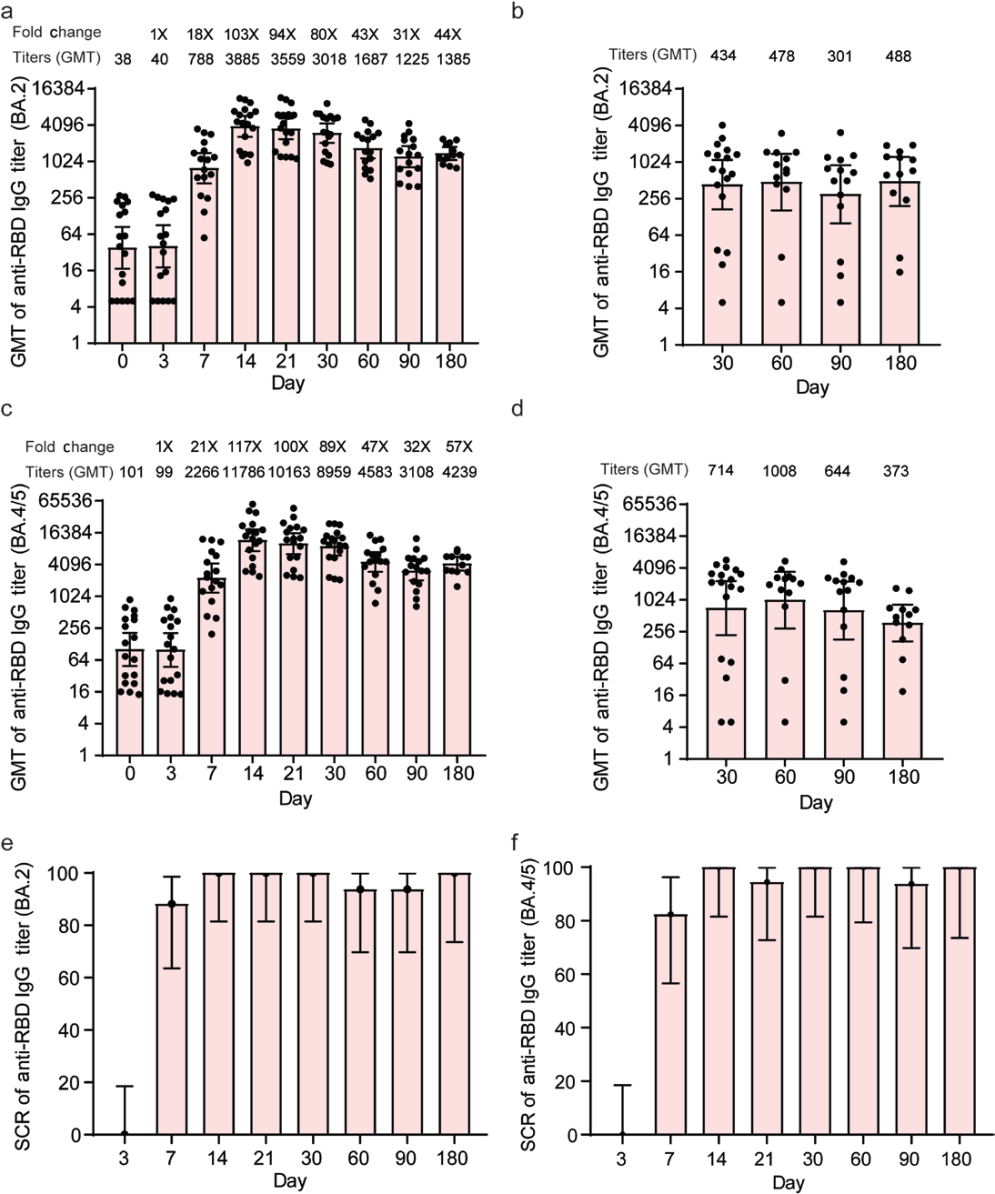
RBD-binding antibodies to Omicron BA.2 and BA.4/5 of SARS-CoV-2. GMTs of RBD-binding IgG titers against Omicron BA.2 in SYS6006 group (a) and convalescent group (b). GMTs of RBD-binding IgG titers against Omicron BA.4/5 in SYS6006 group (c) and convalescent group (d). SCR of RBD-binding IgG titers against Omicron BA.2 (e) and Omicron BA.4/5 (f). Data are GMT (95% CI). Error bars indicate 95% CIs. RBD: receptor binding domain of spike glycoprotein.

Compared with baseline (day 0), SYS6006 boosting vaccination induced significant increases in RBD-binding immunoglobulin G (IgG) titers against Omicron BA.2 variants of SARS-CoV-2 (Fig. 2a) since the day 7 after the vaccination. The geometric mean titers (GMTs) of RBD-binding IgG against Omicron BA.2 before booster were 38 (95% confidence interval (CI) = 17, 84). The titer peaked at day 14 post-vaccination was 3885 (Fig. 2a, *P* < 0.0001). The geometric mean fold increases (GMIs) of IgG titers against Omicron BA.2 were 103-folds at day 14. For convalescent patients, the GMTs of RBD-binding IgG titers against Omicron BA.2 were not different among day 30, 60, 90, and 180 post-confirmed positive, with the GMTs of 434, 478, 301, and 488 respectively (Fig. 2b). In the SYS6006 group, the GMTs of RBD-binding IgG on day 30 and 180 were 7.0- and 2.8-folds of that in the convalescent group, respectively.

GMTs of RBD-binding IgG titers against Omicron BA.4/5 before booster were 101 (95% CI = 49, 208). These GMTs of RBD-binding IgG titers against Omicron BA.4/5 increased to 11786, 8959, 4583, and 3108 at day 14, 30, 60, and 90, respectively, with *P* values < 0.0001 (Fig. 2c). The GMIs of IgG titers against RBD of Omicron BA.4/5 on day 14 were 117-folds (*P* < 0.0001). For convalescent patients, the GMTs of RBD-binding IgG titers against Omicron BA.4/5 were not different among day 30, 60, 90, and 180 post-confirmed positive, with the GMTs of 714, 1008, 644, and 373, respectively (Fig. 2d). The GMTs of RBD-binding IgG against Omicron BA.4/5 on day 30 and 180 were 12.5- and 11.4-folds in the convalescent group, respectively. The seroconversion rates (SCRs) of RBD-binding IgG titers against the Omicron BA.2 and BA.4/5 were >80% until day 90 post SYS6006 vaccination (Fig. 2e and f). Therefore, heterologous boosting with SYS6006 elicited significantly higher RBD-specific IgG titers than that in convalescent patients.

### Neutralizing antibody responses

**Figure 3:**
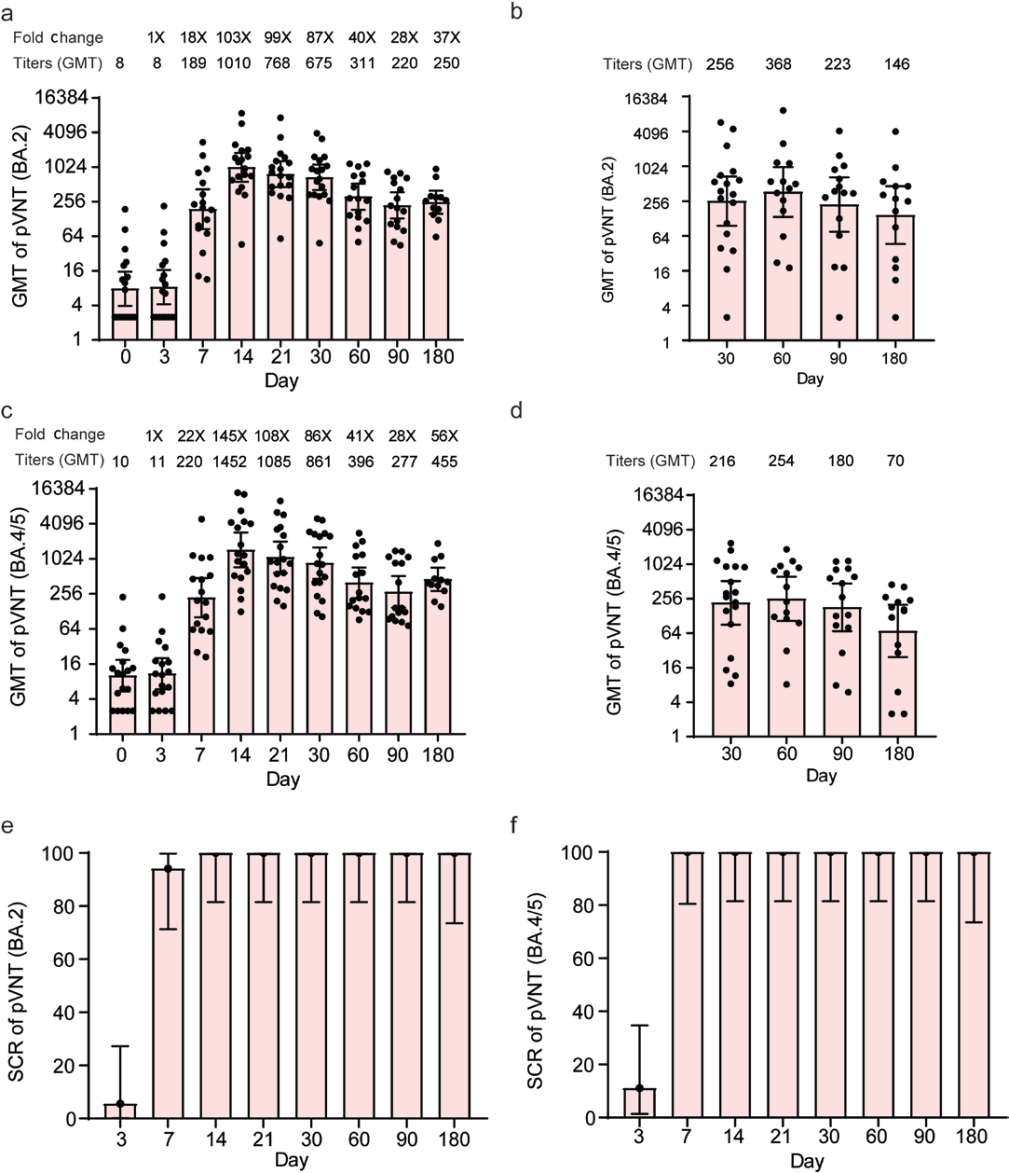
Neutralizing antibodies to SARS-CoV-2 pseudovirus (Omicron BA.2 and BA.4/5) before and after boosting. GMTs of neutralizing antibody titers against Omicron BA.2 in SYS6006 group (a) and convalescent group (b). GMTs of neutralizing antibody titers against Omicron BA.4/5 in SYS6006 group (c) and convalescent group (d). SCR of neutralizing antibody against Omicron BA.2 (e) and Omicron BA.4/5 (f). Data are GMT (95% CI). Error bars indicate 95% CIs. pVNT: pseudovirus neutralization titer.

**Figure 4:**
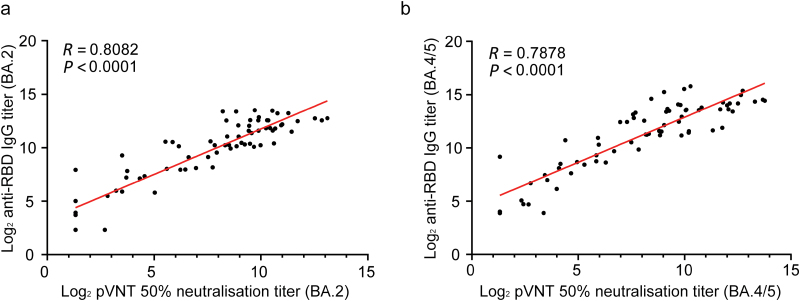
Correlations between neutralizing antibodies and RBD antibodies against Omicron BA.2 and BA.4/5 from day 3 to 21 post-vaccination. (a) Omicron BA.2. (b) Omicron BA.4/5. *R*: correlation coefficient of linear regression.

Consistent with the RBD-binding IgG titers, both heterologous boosters induced significant increases in neutralizing antibody titers against Omicron BA.2 of SARS-CoV-2 (Fig. 3a). SYS6006 induced significantly higher neutralizing antibody titers than that did by convalescent patients. The peak of neutralizing antibodies was observed at day 14 post-booster vaccination with GMT of 1010 (Fig. 3a). For convalescent patients, the GMTs of neutralizing antibody titers against Omicron BA.2 were 256, 368, 223, and 146 at day 30, 60, 90, and 180, respectively (Fig. 3b). The GMIs of neutralizing antibodies against Omicron BA.2 in the SYS6006-20 μg group were 126-folds and remained elevated until day 90 (*P* < 0.0001) (Fig. 3a). The neutralizing antibodies against Omicron BA.4/5 variants showed a similar trend as that against Omicron BA.2, showing significantly higher neutralizing antibodies than those in the convalescent patients’ group (Fig. 3c and d). The SCR maintained over 80% in the SYS6006 group (Fig. 3e and f). These data indicated that heterologous boosting with SYS6006 elicited significantly higher neutralizing antibodies than those in convalescent patients. Correlations between neutralizing antibodies and RBD antibodies by vaccine regimens post-vaccination were evaluated. As shown in Fig. 4, RBD-binding antibody titers positively correlated with neutralizing antibody titers, with correlation coefficients ranging from 0.7878 to 0.8082.

### Vaccine-induced T-cell responses

**Figure 5:**
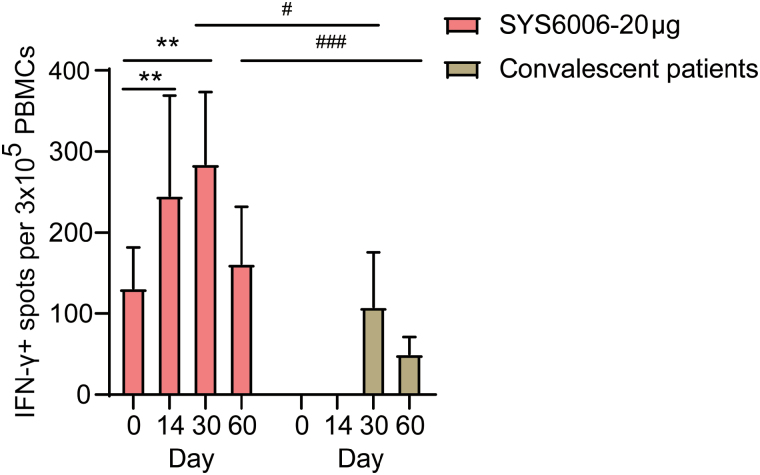
Vaccine-induced T-cell responses. Positive cells excreting specific cytokines (IFN-γ) by ELISpot assay at day 0, 14, 30, and 60 post-vaccination. Data are the geometric mean (95% CI) of positive spot counts per 3 × 10^5^ PBMCs/well. Convalescent patients were in the cabin hospitals and were not able to complete the blood drawing process at day 14 and 21 post COVID-19 infection. ^**^P < 0.01 compared with day 0, ^#^*P* < 0.05, ^###^*P* < 0.001 compared with convalescent patients.

Enzyme-linked immuno-spot (ELISpot) assays against the SARS-CoV-2 RBD using peripheral blood mononuclear cells (PBMCs) were conducted. SARS-CoV-2 specific T-cell responses were measured by detecting the production of IFN-γ. The geometric mean (95% CI) of IFN-γ^+^ spots were 245 (95% CI = 163, 369) at day 14 post-vaccination, and 285 (95% CI = 217, 374) at day 30 post-vaccination compared with 132 (95% CI = 96, 181) before the booster (*P* < 0.01, Fig. 5). Convalescent patients were in the cabin hospitals and were not able to complete the blood drawing process at day 14 and 21 post COVID-19 infection. Participants receiving the SYS6006 had a higher level of IFN-γ^+^ spots than those in the convalescent patients (*P* < 0.05, Fig. 5).

## Discussion

This open-labeled and randomized Phase 1 trial was designed to evaluate the safety and immunogenicity of a modified COVID-19 mRNA vaccine, SYS6006, as a fourth-dose heterologous booster in healthy adults. The primary endpoint of this trial was titers of anti-RBD antibodies of the Omicron strain (BA.2 and BA.4/5) in serum; titers of neutralizing antibodies against pseudovirus the Omicron strain (BA.2 and BA.4/5). The secondary endpoint was the incidence of adverse events within 30 days after the boosting. The exploratory endpoint was the cellular immune responses (IFN-γ). The results indicated that SYS6006 was safe and highly immunogenic. The RBD-binding antibodies and neutralizing antibodies against different strains of SARS-CoV-2 (Omicron BA.2 and Omicron BA.4/5) were consistently elevated in the SYS6006-20 μg group until day 180 post-vaccination. The GMTs of RBD-binding IgG and neutralizing antibodies in the SYS6006 group were significantly higher than those in the convalescent samples. Moreover, SYS6006 boosted strong T-cell responses, with significant induction of IFN-γ^+^ spots.

Injection site pain was the most common local adverse event for SYS6006, similar to the trial results of BNT162b1 [34] and mRNA-1273 [35]. In terms of systemic adverse events, the booster dose of BNT162b1 [34] and mRNA-1273 [35] resulted in fever, headache, fatigue, and malaise, which was also consistent with our data. Meanwhile, the incidence of Grade 3 fever of BNT162b1 at 30 μg was 17% (4/24) in the young Chinese participants [36], while no Grade 3 fever occurred in the SYS6006 group. Besides, the trial results of BNT162b2 and mRNA-1273 showed that the occurrence of Grade 3 fever varied among different age groups [37] and different stages of clinical trials (Phases 1 and 3) [27, 34, 36]. Notably, no decrease in lymphocyte and neutrophil counts was observed in the SYS6006 group, which differed from the results of BNT-162b1 [36] but was similar to the results of mRNA-1273 [35]. Therefore, SYS6006, a modified COVID-19 mRNA vaccine was safe and tolerable in young Chinese adults. No Grade 3 fever or serious adverse events occurred in this study. Given the small sample size of this study with one dose group, more studies were needed for older people in the Phase 3 studies.

The emergence and dominance of SARS-CoV-2 variants have led to reduced waning immunity and efficacy due to reducing cross-reactivity of immune responses to the differentiated VOC [38–40]. Even after the third dose of COVID-19 vaccines, the reduced efficacy resulted in continuous infection cases [41, 42]. It is worth investigating newly developed vaccines [43, 44]. From day 14 to 180, the SYS6006 group had a significantly higher GMTs of binding-IgGs and neutralizing antibodies against BA.2 and BA.4/5 strain of SARS-CoV-2. Besides, more than half of Chinese people had taken a three-dose inactivated vaccine regimen (COVILO or CoronaVac) and it was necessary to evaluate the immunogenicity of a fourth booster dose in this context [45]. Our findings were in line with those of previous studies that booster doses of vaccine-elicited long-lasting humoral immune responses [46]. Besides, SYS6006 elicited cross-reactive neutralizing antibodies against the emerging variants by stimulating neutralizing antibody responses that demonstrated activity against pseudoviruses of the variants of concern (Omicron BA.2 and BA.4/5). Studies have shown a strong correlation between high titers of anti-spike IgG and a lower occurrence rate of infections, indicating that higher IgG titers led to greater protection [47, 48]. The serum samples of convalescent patients were widely used as a comparator of humoral responses [36]. BNT162b1 elicited SARS-CoV-2–neutralizing antibodies, which were similar to the MGT of a panel of SARS-CoV-2 convalescent serum samples. In addition, a strong correlation between neutralizing antibodies and RBD-binding IgG suggested that IgG titers may indicate protection [47]. The RBD-binding antibody and neutralizing antibody titers of SYS6006 were found to be significantly higher from day 14 to 60 post-boosting than that of convalescent serum samples. The results were encouraging and made it worth to explore the protection of SYS6006 against COVID-19 infection in the future clinical studies.

Although attention has been paid to vaccine-induced humoral immune responses, there is compelling evidence that cellular responses contribute to the recovery from both infection and long-term immunity [49, 50]. In the current study, the Th1 (IFN-γ) cellular response induced by SYS6006 was elevated after the booster vaccination. The IFN-γ^+^ spots were significantly higher on day 14 and 30. Since this is the fourth dose of boosting vaccination, the IFN-γ response to the SARS-CoV-2 spike peptide pool preexisted at day 0. Besides, the cellular response was stronger than that in the convalescent patients on day 30 and 60. The results were in line with the results of the third dose of an mRNA vaccine (BNT162b2 or mRNA-1273) [7, 50], showing markedly increased cellular immune responses to the SARS-CoV-2 G614 strain and potentially to all major VOC, including the currently circulating Delta and Omicron variants. Indeed, age-related differences in cellular immune responses existed in both vaccination and SARS-CoV-2 infections, and further studies are needed in different age groups and different infection histories [51, 52].

This study has limitations. First, the convalescent group was recruited in the cabin hospital during the Shanghai COVID-19 lockdown on June 2022. The vaccination history and the strain of SARS-CoV-2 were unable to retrieve. The strain, that the convalescent group was infected with, was presumably Omicron BA.2 according to the reference [33]. Second, the new Omicron substrains (BQ.1.1 and XBB) had stronger immune escape capacity than the early variant strains, but they were not evaluated in this Phase 1 study. Instead, the neutralizing effect against BQ.1.1 and XBB variants were evaluated in the Phase 3 studies. Since SYS6006 has been approved for emergence use in China in March 2023, the data on the new Omicron substrains (BQ.1.1 and XBB) will be disclosed. Finally, we only investigated the immunogenicity and safety of the heterologous boosting with SYS6006, not its effectiveness against COVID-19 infection. Although the high serum neutralizing antibody titers were confirmed, they might not provide protection against the severe COVID-19 symptoms. Additional trials with longer follow-up periods are needed to fulfill the gap.

In conclusion, a modified mRNA vaccine SYS6006 was safe and highly immunogenic as a heterologous booster in healthy Chinese adults. The strong enhancement of antibody titers after heterologous boosting is encouraging, along with the broad-spectrum neutralizing activity of these antibodies against variants of concern, such as Omicron BA.2, and BA.4/5. The titers in the SYS6006 group were higher than those in the convalescent patients. Our findings suggest the flexible use of the mRNA vaccine as a booster regimen, which could accelerate the end of the pandemic.

## Materials and methods

### Study design

We conducted an open-labeled Phase 1 trial to assess the immunogenicity of heterologous booster immunization with SYS6006 in healthy participants (Group A). Moreover, we enrolled convalescent COVID-19 patients (Group B) for comparison of immunogenicity with those in the booster group. The trial protocol and informed consent forms were reviewed and approved by the Ethics Committee of Shanghai Xuhui Central Hospital affiliated with Fudan University. Written informed consent was obtained from each participant before inclusion. This trial was registered with the Chinese Clinical Trial Registry (ChiCTR) website (ChiCTR2200061168) and conducted following the principles of the Declaration of Helsinki, ICH Good Clinical Practice guidelines, and local guidelines. CSPC Pharmaceutical Group Co., Ltd provided the SYS6006 COVID-19 mRNA vaccine (for research use only) but had no role in the trial design, data acquisition, analysis, or interpretation.

### Participants

For Group A, participants were recruited at Shanghai Xuhui Central Hospital. Healthy adults, men and women, <70 years of age, who had completed a three-dose series of inactivated SARS-CoV-2 vaccine (CoronaVac or COVILO) for more than 6 months, were recruited for eligibility screening. Participants received a comprehensive physical examination and inspection, including vital signs, laboratory tests (blood routine, blood biochemistry, urine routine, and coagulation), a 12-lead electrocardiogram, and chest radiography. Investigators verified the vaccination record and checked the medical history of each participant. Key exclusion criteria included: history of infection with SARS-CoV-2 or suspected cases; history of traveling to high outbreak areas or regions outside of China; history of hypersensitivity to acetaminophen or vaccination; history of thrombocytopenia and any coagulation disorder; abnormalities in health examination; severe diseases with atypical clinical manifestations; pregnancy and lactation. For Group B subjects, who have been confirmed positive for SARS-CoV-2 infection within the past 4 weeks and recovered before the screening were enrolled. A detailed list of the inclusion and exclusion criteria was provided on the ChiCTR registration website (ChiCTR2200061168).

### Randomization and masking

This was an open-labeled Phase 1 clinical trial. Healthy participants were enrolled for the fourth dose of SYS6006 while convalescent COVID-19 patients were enrolled for the collection of serum samples. Therefore, no randomization and masking were used. Eligible participants who had completed the three-dose homologous schedule of inactivated SARS-CoV-2 vaccine for over 6 months were enrolled to receive a booster dose of SYS6006. As this was an open-labeled study, both participants and clinical investigators were aware of the treatment allocations in this trial.

### Outcomes

The primary endpoint of this trial was titers of anti-RBD antibodies of the Omicron strain (BA.2 and BA.4/5) in serum; titers of neutralizing antibodies against pseudovirus of the Omicron strain (BA.2 and BA.4/5). The secondary endpoint was the incidence of adverse events within 30 days after the boosting. The exploratory endpoint was the cellular immune responses (IFN-γ) measured by ELISpot assay, of which the results were expressed as the number of positive spots per 300,000 cells.

### Procedures

Participants of Group A were injected with the SYS6006 (CSPC Pharmaceutical Group Co., Ltd), which was an mRNA SARS-CoV-2 vaccine and contained 30 µg (0.3 mL)/vial. A trained designated nurse was responsible for the vaccine preparation, by extracting 0.2 mL (20 µg) of vaccine from each vial of SYS6006 by using standard syringes and needles. The vaccine was intramuscularly administered in the deltoid muscle. After the booster vaccination, all participants were hospitalized at the clinical research center for at least 24 h. Vital signs, physical examination, 12-lead electrocardiogram, blood routine, urine routine, blood biochemistry, and coagulation function were tested on the third day after vaccination. Solicited injection site events included pain, pruritus, blush, swelling, rash, induration, and cellulitis; while systemic events included fever, diarrhea, fatigue, nausea, anorexia, vomiting, headache, cough, arthralgia, etc. Solicited adverse events within 14 days were collected by investigators. Unsolicited adverse events within 30 days were reported by the participants and were also collected by investigators. Special attention was paid to the occurrence of vaccine-enhanced disease (VED) phenomena during this study. Adverse events were observed and the severity was graded according to the “Guidelines for the Classification of Adverse Events in Clinical Trials of Prophylactic Vaccines” issued by the National Medical Products Administration (NMPA) of China. Adverse events not included in the guidelines were assessed according to CTCAE 5.0. For Group A participants, safety assessment was not conducted.

Serum samples were collected at baseline (day 0) and on day 3, 7, 14, 21, 30, 60, 90, and 180 after booster vaccination to facilitate measurement of specific binding IgG antibody responses to RBD of SARS-CoV-2 spike glycoprotein and neutralizing antibody responses to pseudovirus. PBMC samples were collected at baseline (day 0) and on day 14, 30, and 60 post-vaccinations for IFN-γ specific T-cell responses.

For Group B participants, serum samples were collected on day 14, 21, 30, 60, 90, and 180 after confirmed positive for SARS-CoV-2 for measurement of specific binding IgG antibody responses to RBD of SARS-CoV-2 spike glycoprotein and neutralizing antibody responses to pseudovirus. PBMC samples were collected at baseline (day 0), and on day 14, 30, and 60 post-vaccinations for IFN-γ specific T-cell responses.

### Immunogenicity assessment

Titers of spike RBD-specific binding antibodies were assessed using an indirect enzyme-linked immunosorbent assay (ELISA). Omicron strain BA.2 (Cat. No. DD9104) and Omicron strain BA.4/5 (Cat. No. DD9114) assay kits were obtained from Nanjing Vazyme Biotech Co., Ltd. Briefly, serum samples were serially diluted in a 2-fold manner before minimal required dilution (MRD) of 1:10. Then, diluted samples were added into spike RBD protein coated microplate and incubated at 37°C for 30 min. A horseradish peroxidase-conjugated secondary antibody was then added to the plate and incubated at 37°C for 30 min. Data collection was performed using a SpectraMax M2 reader (Molecular Device) to measure the optical density (OD) at 450 and 610 nm with the SoftMax Pro GxP Software (version 7.1.1). The two-points adjacent to the assay cutoff value were chosen for linear fitting to determine the sample titer, and the sample dilution corresponding to the cutoff value served as the titer value. If the OD_450 nm_–OD_610 nm_ value was less than the cutoff at an initial dilution (1:10 for Omicron BA.2 and BA.4/5), half of the limit of quantification was reported.

Titers of neutralizing antibodies against pseudovirus of SARS-CoV-2 were determined using a microneutralization assay with the recombinant vesicular stomatitis virus vector (rVSV) coronavirus pseudovirus (rVSV-SARS-CoV-2) and the Vero cells highly expressing ACE2 receptors (Nanjing Vazyme Biotech Co., Ltd). Pseudoviruses of BA.2 (Cat. No. DD1502) and BA.4/5 (Cat. No. DD1502) were obtained from Nanjing Vazyme Biotech Co., Ltd. In brief, serum samples were 3-fold serially diluted from 1:5 to 1:1215 (Omicron strain BA.2 and BA.4/5) using DMEM (Opm Biosciences). Serum dilutions were then mixed with virus solutions to achieve 650 TCID_50_/well. The serum–virus mixture was incubated at 37°C for 1 h, and then Vero cells of 4 × 10^4^/well were added to 96-well plates. After overnight culturing, the luciferase substrate was added and the data collection was performed using an Envision microplate reader (Perkin & Elmer). The neutralizing titer was the reciprocal of the highest sample dilution that protects at least 50% of cells from rVSV infection. If no neutralization reaction was observed at the initial serum dilution, half of the limit of quantification was reported.

SARS-CoV-2 S-specific T-cell responses after vaccination (IFN-γ) were assessed using an *ex vivo* ELISpot Assay. Briefly, PBMCs were isolated by Ficoll-Paque PLUS (Cytiva) density gradient centrifugation on baseline and day 7, 14, 30, and 60 after the boosting vaccination. The PBMCs were cryopreserved before analysis. T_H_1-secreted cytokines (IFN-γ, Cat. No. 3420-2AST-10, Mabtech) were detected by the ELISpot assay. Tests were performed with the positive control (PMA, 25 ng/mL in 100 μL). Peptide pools of SARS-CoV-2 covering the full-length spike glycoprotein were prepared at a concentration of 400 ng per well, and 300,000 cells per well were added to the plate. PBMCs were stimulated for 18–24 h with the peptide pools. Bound IFN-γ^+^ points were visualized using a secondary antibody directly conjugated with alkaline phosphatase followed by incubation with 5-bromo-4-chloro-3ʹ-indolyl phosphate and nitro blue tetrazolium substrate. Plates were scanned using an ES-15 ELISPOT Reader (Fosun Diagnostics).

### Statistical analysis

The sample size for the trial followed the sample size requirements of NMPA and Chinese Technical Guidelines for Clinical Trials of Vaccines, which required at least 15–30 participants in a Phase 1 trial. We assessed the safety endpoints in all the participants who received the boosting vaccination. The IgG against the RBD of SARS-CoV-2 was presented as GMTs, GMIs, and SCR, defined as the proportion of participants with at least a 4-fold increase after booster vaccination. GMTs and GMIs were calculated with the two-sided 95% CI, based on the *t*-distribution of the log-transformed titers. Shapiro–Wilk test was used for normality determination of the log-transformed data. Analysis of variance was used to compare normally distributed data across the groups, and the Kruskal–Wallis test was used for non-normal distributed data. Data below the detection limit were assigned a value half of the threshold. Categorical data, including the incidence of adverse reactions and SCRs, were presented as counts and percentages with 95% CI calculated using the Clopper–Pearson method. χ^2^ test or Fisher’s exact test was used to compare categorical data. Spearman correlation analysis of the association between log-transformed antibody neutralization titers and RBD-binding IgG titers in serum was done. Two-sided *P*-values of <0.05 were considered significant without multiplicity adjustment. Statistical analyses were performed using SAS (version 9.4) or GraphPad Prism 8.0.1.

## Data Availability

We support data sharing of the individual de-identified participant data that underlie the results reported in this article. Data will be available when the trial is completed. Clinical study reports and study protocol with any amendments will be shared upon request with the corresponding author.
